# Preparation of Palladium(II) Ion-Imprinted Polymeric Nanospheres and Its Removal of Palladium(II) from Aqueous Solution

**DOI:** 10.1186/s11671-017-2349-5

**Published:** 2017-11-06

**Authors:** Hu-Chun Tao, Yi-Han Gu, Wei Liu, Shuai-Bin Huang, Ling Cheng, Li-Juan Zhang, Li-Li Zhu, Yong Wang

**Affiliations:** 1 Key Laboratory for Heavy Metal Pollution Control and Reutilization, School of Environment and Energy, Peking University Shenzhen Graduate School, Shenzhen, 518055 China; 2 Guangdong Provincial Key Laboratory of Nano-Micro Material Research, School of Chemical Biology & Biotechnology, Peking University Shenzhen Graduate School, Shenzhen, 518055 China

**Keywords:** Palladium(II), Ion-imprinted polymeric nanospheres, Functional monomer, Adsorption

## Abstract

Three kinds of functional monomers, 4-vinylpridine(4-VP), 2-(allylthio)nicotinic acid(ANA), and 2-Acetamidoacrylic acid(AAA), were used to synthetize palladium(II) ion-imprinted polymeric nanospheres (Pd(II) IIPs) via precipitation-polymerization method in order to study the effects of different functional monomers on the adsorption properties of ion-imprinted materials. The results of UV spectra in order to study the interaction between template ion PdCl_4_^2−^ and functional monomers showed that there were great differences in structure after the template reacted with three functional monomers, 4-VP and ANA caused a large structural change, while AAA basically did not change. Further results on the adsorption performance of Pd(II) IIPs on Pd(II) confirmed 4-VP was the most promising candidate for the synthesis of Pd(II) IIPs with an adsorption capacity of 5.042 mg/g as compared with ANA and AAA. The influence of operating parameters on Pd(II) IIP’s performance on Pd(II) adsorption was investigated. There was an increase in the adsorption capacity of Pd(II) IIPs at higher pH, temperature, and initial concentration of Pd(II). The results of multi-metal competitive adsorption experiments showed that Pd(II) IIPs had selectivity for Pd(II). An adsorption equilibrium could be reached at 180 min. Kinetic analysis showed that the adsorption test data fitted best to the pseudo-second order kinetic model, and the theoretical equilibrium adsorption capacity was about 5.085 mg/g. The adsorption isotherms of Pd(II) by Pd(II) IIPs agreed well with the Freundlich equation, suggesting a favorable adsorption reaction under optimal conditions. These results showed that Pd(II) IIPs have potential application in the removal of Pd(II) from aqueous solutions and may provide some information for the selection of functional monomers in the preparation of Pd(II) IIPs.

## Background

Palladium with unique physical and chemical properties is widely used in electroplating materials, catalysts, dental alloy, and brazing alloy [1, 2]. With the increasing application of palladium in various fields, a considerable amount of wastewater containing palladium can be produced. The discharge of the wastewater containing palladium can not only cause serious waste of resources, but also cause great pollution to the environment and do harm to human health [3–6]. Separation and enrichment in some studies can solve these problems, the common enrichment and separation methods include co-precipitation [7], adsorption [8] and ion exchange [9], liquid-liquid extraction [10] and solid phase extraction [11], micro-liquid extraction [12], and cloud point extraction [13]. There are many studies on the enrichment and separation of palladium [14–18]; among them, the adsorption method is widely used in many ways because it is simple, convenient, and efficient. The performance of common adsorbents include activated carbon, however, is not highly selective for sequestering palladium ions from aqueous solutions containing several metals. Therefore, development of palladium separation material with high selectivity for the removal, recovery, and recycling of palladium ions from the waste solution is of great significance.

Ion-imprinted polymeric nanospheres with high selectivity for the separation of metals from aqueous solution compared with other common adsorbents has become one of the research hotspots in recent years [19–23]. In the preparation of ion-imprinted polymeric nanospheres, the stability of chelates formed by functional monomers with different functional groups and metal ion by ion bond or coordination bond is depending on the interaction strength between the functional monomer and metal ions, the stronger the interaction, the stronger the ability of ion-imprinted polymer to chelate metal ions and the greater the adsorption performance. So it is important to select functional monomers [24].

Many studies used 4-VP as the functional monomer in the preparation of Pd(II) IIPs, while few studies have involved the comparison of 4-VP with other functional monomers [25–30]. In this study, two kinds of uncommon functional monomers ANA and AAA were used to compare with common 4-VP. The interaction between PdCl_4_^2−^ and functional monomer was analyzed by UV full wavelength scanning. Then, the best functional monomer was selected by comparing the adsorption effect of Pd(II) IIPs corresponding to three functional monomers on palladium(II). Through batch adsorption experiments, the adsorption performance of Pd(II) IIPs for palladium(II) ions in aqueous solutions was evaluated. Various characterization means of FTIR, SEM, and TGA were utilized to further explore the corresponding mechanism of Pd(II) adsorption onto Pd(II) IIPs.

## Methods

### Materials

The following chemicals K_2_PdCl_4_, 4-vinyl pyridine (4-VP, 96%), 2-allyl sulfhydryl nicotinic acid (ANA, 98%), 2-acetamidoacrylic acid (AAA, 99%), and ethylene glycol dimethacrylate (EGDMA, 98%) were purchased from Alfa company in the USA. Azo isobutyronitrile (AIBN, 99%) was purchased from Shanghai zhongfugang Co. Ltd. Palladium single element standard solution was purchased from Chinese national standard material network. All chemicals were in analytical reagent grades and used without further modification. Ultrapure water was used to prepare all the solutions. All of the glassware was cleaned and rinsed with Milli-Q water and then dried in an oven overnight before using.

### Preparation of Palladium(II) Ion-Imprinted Polymeric Nanospheres

The palladium(II) ion-imprinted polymeric nanospheres were synthesized by precipitation-polymerization method. In the precipitation procedure, Pd(II) IIPs were prepared according to the ratio of template (PdCl_4_^2−^), functional monomers (4-VP, ANA, AAA) and cross-linking monomer at 1:4:40. In the polymerization procedure, ethylene glycoldimethacrylate (EGDMA) was used as the cross-linking monomer, the polymerization mixture also included 2,2-azobisisobutyronitrile (AIBN, initiator) and methanol (Porogen). The detailed operation is as follows:

Firstly, 0.1 mmol K_2_PdCl_4_ was dissolved in 20 mL of methanol in a 50 mL glass flask, then 0.4 mmol 4-VP was added and stirred in a thermostatic oscillator at 25 °C for 3 h. Secondly, 4 mmol of EGDMA and 36.13 mg AIBN were added to the glass flask, and the obtained solution was transferred into thick wall pressure bottles. The oxygen of the sample solution was removed by bubbling nitrogen gas through the sample for 10 min. Polymerization was performed in a water bath at 60 °C for 24 h under stirring at 180 rpm. The prepared polymer was washed several times with 1:4 (*v*/*v*) methanol/water to remove the unreacted materials, and then palladium ions (PdCl_4_^2−^) was leached from the polymer material by stirring with 4 × 50 ml of 1:1 HCl for 24 h until the washing solution was free from palladium ions. Finally, it was washed with deionized water until it reached a neutral pH. The polymers were dried under vacuum in a desiccator. In the same way, the non-imprinted polymers (NIPs) were prepared but without doping palladium ions.

### Characterizations

Ultraviolet visible spectrophotometer (UV-2600, Shimadzu, Japan) was used to analyze the interaction between PdCl_4_^2−^ and functional monomer. Field-emission scanning electron microscope (SU8040, Hitachi, Japan) was used to observe the morphology changes of ion-imprinted polymer before and after elution and non-ion-imprinted polymer. Fourier transform infrared (FTIR) spectra of the Pd(II) IIPs before and after elution, and NIPs were analyzed with FTIR Spectrometer (Nicolet 6700, Thermo-Nicolet, USA) with KBr pellets in the range of 4000~400 cm^−1^. Brunauer, Emmett, Teller’s test (BET, TriStarII3020) was used to analyze the specific surface area. Thermogravimetry analysis (TGA) was performed using Netzsch STA-409PC (Germany) from 313 to 873 K under a dried nitrogen atmosphere, and the heating rate was 10 K/min.

### Batch Adsorption Experiments

The concentration of Pd(II) was determined by flame atomic absorption spectrophotometry (FAAS, TAS-990, Persee, China). All batch adsorption experiments were performed using a thermostatic oscillator at 180 rpm with 10 mg adsorbent in a 50 mL plastic centrifuge tube containing 10 mL metal solution. Samples were taken in triplicate for all batch experiments. The effect of temperature on the adsorption of Pd(II) onto Pd(II) IIPs was evaluated at 15, 25, 35, 45, and 55 °C. Four interfering metal ions include Pt(II), Zn(II), Cu(II), and Ni(II) with an initial concentration of 10 mg/L were chosen to study the effect of multiple metals on adsorption of Pd(II).

Isotherm adsorption experiments were carried out with a constant dosage of adsorbents and varying concentration of Pd(II) in the range of 1~80 mg/L at 25 °C (pH 2). Adsorption kinetic experiments were conducted by collecting solution at predetermined time intervals (sampling time was set to 5, 8, 10, 15, 20, 25, 30, 40, 60, 120, 180, 240, and 300 min) and analyzing the final metal concentration in the aqueous solutions.

The percentage of Pd(II) removal and the adsorption capacity of the Pd(II) IIPs for Pd(II) ions can be calculated according to the following equations:

1 $$ r=\left({c}_0-{c}_e\right)/{c}_0\times 100\% $$

2 $$ q=\left({c}_0-{c}_e\right)\times V/m $$

in which *r* (%) is the removal efficiency of Pd(II), *q* (mg/g) is the capacity of Pd(II) adsorbed onto the adsorbent of Pd(II) IIPs, *c*_e_ (mg/L) is the concentration of Pd(II) in solution at equilibrium, *c*_0_ (mg/L) is initial concentration of Pd(II) in solution, *V* (mL) is the volume of Pd(II) solution, and *m* (mg) is the mass of adsorbent.

The Langmuir (Eq. 3) and Freundlich (Eq. 4) isotherm models can be mathematically represented by the following equations:

3 $$ \frac{c_e}{q_e}=\frac{1}{bq_m}+{c}_e\frac{1}{q_m} $$

4 $$ \ln {q}_e=\ln {K}_f+\frac{1}{n}\times \ln {c}_e $$

where *q*_e_ is the amount of Pd(II) adsorbed on adsorbents at equilibrium (mg/g), *q*_m_ is the theoretical maximum adsorption capacity of adsorbents under the certain conditions (mg/g), *c*_e_ is the concentration of Pd(II) in aqueous solutions at equilibrium(mg/L), *b* is the Langmuir constant connected to the affinity between the Pd(II) and the adsorbents (L/mg), *K*_f_ is the Freundlich constant related to the adsorption capacity of adsorbents, and 1/*n* is the heterogeneity factor ranging from 0 to 1.

In order to further clarify the dynamics and rate-controlling mechanism for adsorption process, two commonly used kinetics models, i.e., the pseudo-first-order kinetics and the pseudo-second-order kinetics, were utilized to simulate the experimental adsorption data. The pseudo-first-order (Eq. 5) and the pseudo-second-order (Eq. 6) kinetics can be mathematically expressed as:

5 $$ {q}_t={q}_e\left(1-{e}^{\left(-{k}_1t\right)}\right) $$

6 $$ {q}_t=\frac{q_e^2{k}_2t}{1+{q}_e{k}_2t} $$

where *q*_e_ is the amount of Pd(II) adsorbed on adsorbents at equilibrium (mg/g), *t* is the contact time during adsorption process, *q*_t_ is the amount of Pd(II) adsorbed on adsorbents at any time *t* (mg/g), k_1_ is the rate constant of the pseudo-first-order model (min^−1^), and *k*_2_ is the rate constant of the pseudo-second-order model (g/mg min).

## Results and Discussion

### The Optimization of Functional Monomer

**Fig. 1:**
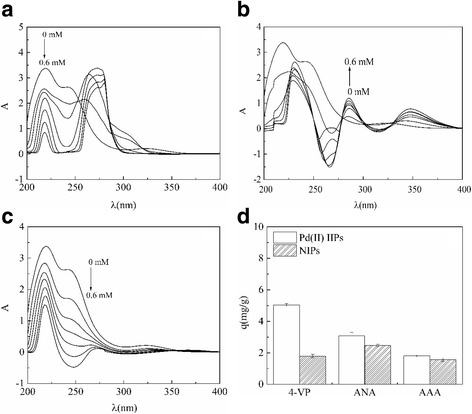
Ultraviolet spectra of the interactions between PdCl_4_^2−^ and (**a**) 4-VP, (**b**) ANA, (**c**) AAA in methanol and (**d**) adsorption properties of Pd(II) IIPs synthesized by different function monomers

The UV spectra of PdCl_4_^2−^ and 4-VP, and ANA and AAA in methanol before and after the interaction are shown in Fig. 1. It can be seen from the figure that PdCl_4_^2−^ has two absorption peaks at 219.4 and 242.4 nm, and the absorption peaks are shifted when different functional monomers are added. When functional monomer of 4-VP is dosed (Fig. 1a), there is a hypochromic effect on PdCl_4_^2−^ appeared at 219.4 and 242.4 nm, and a new absorption peak formed at around 275 nm as a result of hyperchromic effect compared to that at 219.4 nm, suggesting the obvious changes in the structure of PdCl_4_^2−^ and 4-VP in the vicinity of 275 nm. When ANA was added in PdCl_4_^2−^ methanol solution as a functional monomer (Fig. 1b), PdCl_4_^2−^ appeared red shift phenomenon at 219.4 and 242.4 nm, and two new absorption peaks emerged in the vicinity of 285 and 347 nm as compared to the absorption peak at 219.4 nm, the two newly formed peaks can be ascribed to the hypochromic effect, indicating that both PdCl_4_^2−^ and ANA have some difference in their structures in the vicinity of 285 and 347 nm. It can be seen from Fig. 1c that the addition of AAA does not give any red shift or blue shift to the absorption peak of PdCl_4_^2−^ at both 219.4 and 242.4 nm, and there is no new absorption peak, indicating the negligible changing in the structure of PdCl_4_^2−^ and AAA.

In order to further study the adsorption effect of Pd(II) on Pd(II) IIPs and NIPs prepared by 4-VP, ANA, and AAA, the adsorption of Pd(II) on each materials was measured. As shown in Fig. 1d, the amount of Pd(II) ions adsorbed onto Pd(II) IIPs was greater than that onto the corresponding NIPs. Besides, according to the BET method, the surface area of IIPs and NIPs was calculated (Table.1): the surface area of IIPs prepared by 4-VP is 23.74 m^2^/g exceed NIPs (0.46 m^2^/g). It means that a kind of polymeric nanospheres Pd(II) IIPs with a larger surface area was produced after adding the imprinted ion. This observation can be interpreted by the significant differences in the spatial structure of Pd(II) IIPs and NIPs with the same kind of functional monomers. In the process of Pd(II) IIPs formation, due to the addition of imprinted Pd (II) ion, the functional monomer and the Pd(II) ion formed a coordination complex with the imprinting cavities, and the hole of the Pd(II) ion presents a “memory,” which leads to more adsorption amount of Pd(II) ions on Pd(II) IIPs than on NIPs. In addition, Table.1 showed the Pd(II) adsorption capacity of the three kinds of polymers increased with an order as 4-VP > ANA > AAA, indicating that the Pd(II) IIPs prepared by 4-VP were the best. The results were caused by the lone pair electrons of N atoms in the structure of 4-VP that can not only be chelated with metal ions, but also form hydrogen bonds with functional groups such as carboxyl and hydroxyl groups. In addition, the vinyl groups in the structure can react with the cross-linking agent so that the atom N is hung on the chain of the polymer to form a weak alkaline polyelectrolyte.

**Table 1:** Specific area and adsorption capacity of different materials

	IIPs			NIPs
Functional monomer	4-VP	ANA	AAA	4-VP
Adsorption (mg/g)	5.042	3.121	1.804	1.636
Surface area (m^2^/g)	23.7379	20.853	9.7103	0.4604
Adsorption per unit surface area (mg/m^2^)	0.2124	0.1497	0.1858	3.5534

**Fig. 2:**
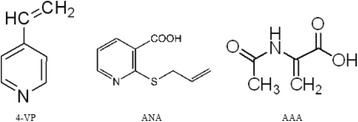
Structural formula of three functional monomers

By comparing the structure of these three kinds of functional monomers (Fig. 2) and the adsorption test results, we found that the adsorption effect of 4-VP containing nitrogen heterocycles was the best, followed by ANA containing both nitrogen heterocyclies and carboxyl groups, and the worst was AAA containing carboxyl groups. Therefore, we speculated that the binding of Pd(II) ions and functional monomers containing nitrogen heterocycles are stronger than that containing carboxyl groups, and the presence of carboxyl groups may weaken the binding of Pd(II) ions and functional monomers containing both nitrogen heterocyclies and carboxyl groups.

The Pd(II) IIPs mentioned in the following studies were all prepared by using 4-VP as the functional monomer.

### Characteristics of Pd(II) IIPs and NIPs

**Fig. 3:**
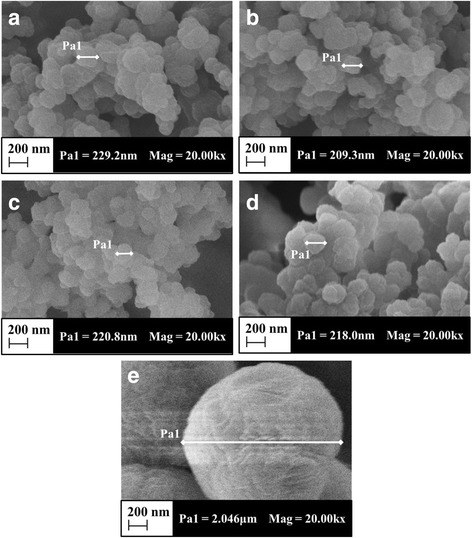
SEM of (**a**) 4-VP - leached IIPs, (**b**)ANA - leached Pd(II) IIPs, (**c**) AAA- leached Pd(II) IIPs, (**d**) 4-VP - Unleached IIPs, (**e**) 4-VP - leached NIPs. (All the images under the same magnification in 20,000X)

The microscopic morphology of leached Pd(II) IIPs, unleached Pd(II) IIPs and NIPs were observed. It can be seen from Fig. 3a, d that there are no changes in the morphology of neither unleached Pd(II) IIPs nor leached Pd(II) IIPs; besides, BET method showed that leached Pd(II) IIPs’ specific surface area (23.74m^2^/g) was similar with the unleached Pd(II) IIPs (22.49m^2^/g), the little difference can be ignored, so it can be concluded that the elution has no effect on the morphology of the Pd(II) IIPs. The surface of the polymer becomes relatively rough after the addition of the PdCl_4_^2−^ template, which is due to the formation of imprinted holes. In comparison, the NIPs exhibit a smoother surface (Fig. 3e) with a much larger particle size of 2 μm than that of unleached Pd(II) IIPs and leached Pd(II) IIPs (around 200 nm) under the same magnification. This finding indicated that the addition of template PdCl_4_^2−^ exerts a great influence on the morphological properties of palladium(II) ion-imprinted polymer.

**Fig. 4:**
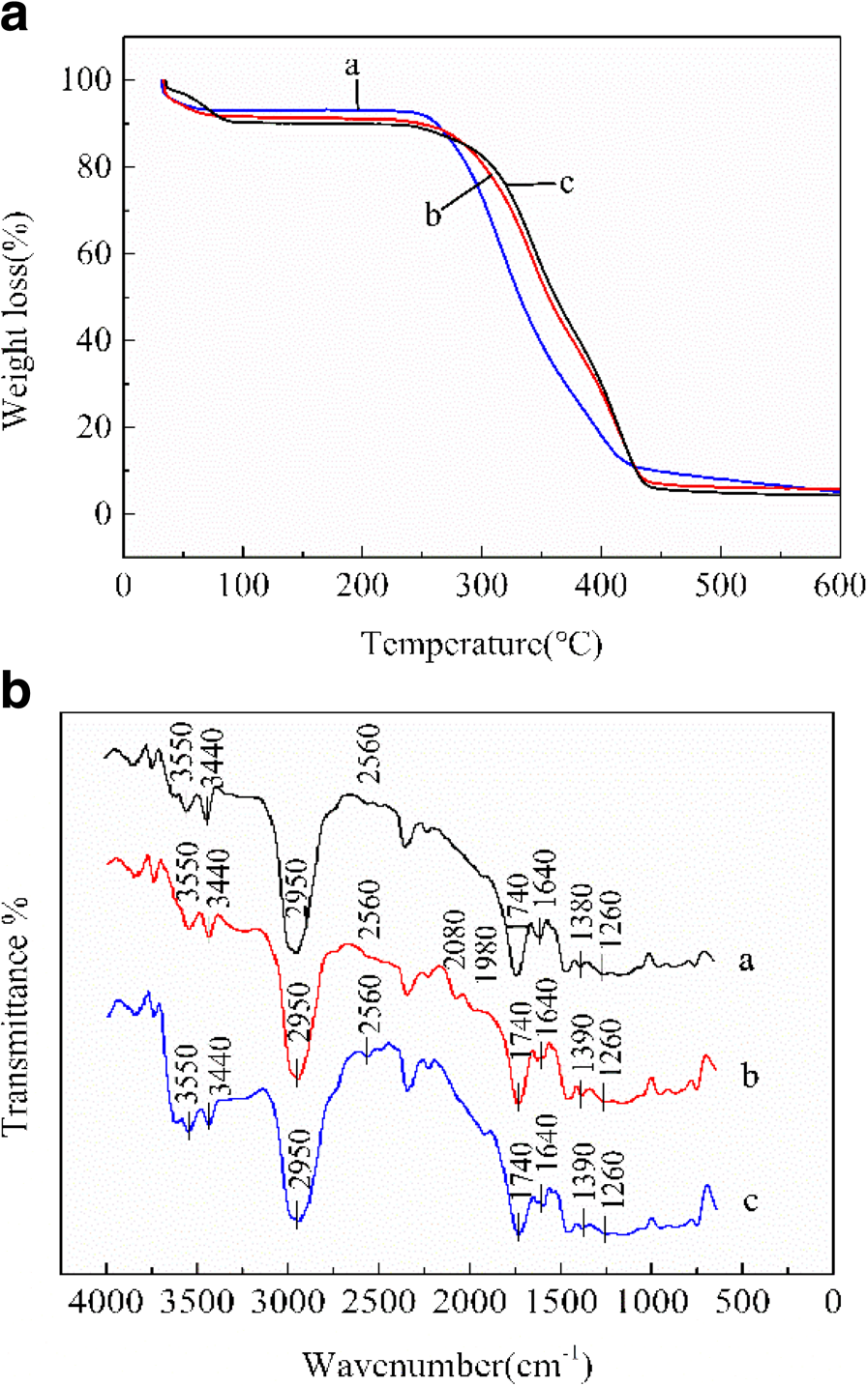
**a** TGA curves of a: unleached Pd(II) IIPs, b: leached Pd(II) IIPs, c: NIPs; (**b**) FTIR spectra of a: unleached Pd(II) IIPs, b: leached Pd(II) IIPs, c: NIPs

Different decomposition behavior can be observed from the thermogravimetric curves of unleached Pd(II) IIPs, leached Pd(II) IIPs, and NIPs (Fig. 4a). At lower temperature of 40~100 °C, the thermal decomposition rate is relatively low. The weight loss is mainly attributed to the evaporation of free and/or bound water molecules. The major composition of samples has not yet started decomposition at the temperature of 100~250 °C. At temperature higher than 250 °C, the samples begin to lose weight rapidly; this is mainly due to the decomposition of organic matters in the polymers with the increasing of temperature. As the temperature elevated to above 440 °C, the organic matters in the polymers have been decomposed completely, reaching a state of thermal stability. When the temperature goes up to 600 °C, the residue mass percentage of unleached Pd(II) IIPs arrive at around 6%. The residual matters are supposed to be mainly comprised of inorganic palladium.

Based on the absorption peaks and bands on FTIR spectra, many functional groups on the surface of absorbent material can be depicted and characterized for unleached Pd(II) IIPs, leached Pd(II) IIPs, and NIPs (Fig. 4b). It can be seen from the figure that the bands observed at 3440 and 1640 cm^−1^ are assigned to the stretching vibration frequency of C-N and -CONH- in amide, respectively. The peaks at 3550, 2950, 2560, 2350, 1740, and 1260 cm^−1^ are attributed to the stretching vibration frequency of −OH, C-H, S-H, C=O in esters and C-O, respectively. There is no shift among these vibrational absorption peaks in the three FTIR curves due to no coordination. As shown in curve b and curve c, the peak attributed to C-N shifted from 1390 to 1380 cm^−1^ after adding template ion Pd(II), indicating the coordination happened between the template ion Pd(II) and functional monomer. In addition, new absorption peaks appeared at 2080 and 1980 cm^−1^ in curve b may be caused by the elution process of Pd(II) which can lead to some changes in groups.

### Batch Adsorption Experiments

**Fig. 5:**
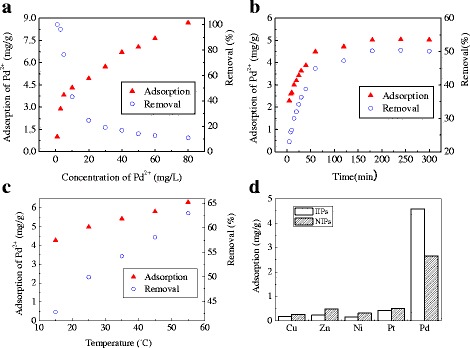
Effects of (**a**) initial concentration, (**b**) contact time, (**c**) temperature and (**d**) multiple metals on adsorption of Pd(II) on Pd(II) IIPs

The effect of the initial concentration of Pd(II) ions on the adsorption capacity of Pd(II) IIPs is shown in Fig. 5a. When the dosage of Pd(II) IIPs is fixed, the adsorption capacity of Pd(II) IIPs for Pd(II) ions increase with the increasing of initial concentration of adsorbate ions, while the removal efficiency decreases accordingly. This is due to the limited adsorption sites provided by Pd(II) IIPs in solution. At lower Pd(II) concentration, the amount of active adsorption sites is abundant to absorb the majority of Pd(II) ions in solution. As the initial concentration of Pd(II) ions increased, however, the available number of active adsorption sites is limited by the fixed dosage of adsorbent. There is no further active sites to combine the excess Pd(II) ions at higher concentration. Accompanied by the gradual saturation of adsorption capacity of Pd(II) IIPs, there is a constant decrease in Pd(II) removal efficiency.

The effect of contact time on adsorption capacity and removal efficiency of Pd(II) IIPs for Pd(II) ions is shown in Fig. 5b. In early test period of 60 min, both the adsorption capacity and removal efficiency of Pd(II) IIPs for Pd(II) increase quickly as the contact time of reaction elongates. At the very beginning of reaction, a large number of binding sites are available on Pd(II) IIPs for Pd(II) adsorption together with the relatively high concentration of Pd(II) ions, there exists a strong driving force to promote the mass transfer of adsorbate ions from bulk solution to the unoccupied binding sites. As a consequence, it is conducive for Pd(II) IIPs acting as an efficient adsorbent to remove heavy metals from wastewater in the first 3 h. However, as the contact time extends, a majority of active sites of Pd(II) IIPs combined with Pd(II) ions and the available active sites decreased. After 180 min, the adsorption capacity and removal efficiency of Pd(II) IIPs remains unchanged and reaches an equilibrium state. Therefore, 180 min was set as the optimal contact time for adsorption process.

Figure 5c represents the effect of operating temperature on adsorption capacity and removal efficiency of Pd(II) IIPs for Pd(II). We found that the adsorption capacity and removal efficiency of Pd(II) IIPs for Pd(II) increases with the increasing of temperature, indicating an endothermic process for the adsorption reaction. Higher temperature is beneficial to promote the adsorption capacity of absorbents. In general, at normal engineering temperature between 25 and 35 °C, Pd(II) IIPs can have a good performance in practical application.

The influence of multiple metals on the adsorption of Pd (II) by Pd(II) IIPs and NIPs is shown in Fig. 5d. In the coexisting system of multiple metals, the adsorption capacity of Pd(II) IIPs and its corresponding NIPs on Pd(II) is the largest, followed by Pt, Zn, Ni, and Cu. The adsorption capacity of Pd(II) IIPs on Pd(II) was 26.7, 21.5, 31.8, and 10.4 times higher than that of Cu(II), Zn(II), Ni(II), and Pt(II), respectively. The results indicated that Pd(II) IIPs was highly efficient and selective for Pd (II). The adsorption capacity of Pd(II) IIPs on Pt(II) was larger than that on Cu(II), Zn(II), and Ni(II), which may because of Pt(II)’s chemical similarity to Pd(II) and competitive adsorption sites compared with other metals. The adsorption capacity of NIPs on Cu(II), Zn(II), Ni(II), and Pt(II) was larger than that of Pd(II) IIPs, whereas Pd (II) is exactly the opposite, indicating that better adsorption effect of Pd(II) IIPs on Pd(II) than that of NIPs is not caused by large specific area but the formation of the recognition adsorption sites for Pd(II) in the preparation process.

### Isothermal and Kinetic Studies

**Fig. 6:**
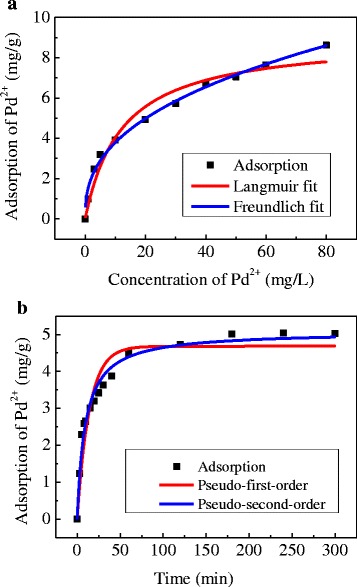
**a** Adsorption isothermal and (**b**) kinetic fitting curves of Pd(II) on Pd(II) IIPs

To explore the adsorption capability of Pd(II) IIPs, two typical adsorption isotherm models, i.e., the Langmuir and Freundlich adsorption isotherms models (Fig. 6a), were utilized to investigate adsorption mechanism. The experimental data are then fitted with the pseudo-first-order and the pseudo-second-order kinetic models (Fig. 6b). The isothermal and kinetic parameters for respective models are summarized in Table 2.

**Table 2:** Isothermal and kinetic parameters of Pd(II) on Pd(II) IIPs

Isothermal models						
Parameter	Langmuir			Freundlich		
	*q*_*m*_ (mg/g)	*b* (L/mg)	*R* ^2^	*K*_*f*_ (mg/g)/(mg/L)^1/n^	*n*	*R* ^2^
Value	9.00	0.11	0.946	1.532	2.538	0.991
Kinetic models						
Parameter	Pseudo-first-order			Pseudo-second-order		
	*k*_1_ (min^−1^)	*q*_*e*_ (mg/g)	*R* ^2^	*k*_2_ (g/(mg min))	*q*_e_ (mg/g)	*R* ^2^
Value	0.071	4.683	0.896	0.02	5.085	0.971

In the isotherm study, the correlation coefficient of the Freundlich isotherm model (*R*^2 = 0.991) is much closer to 1 as compared with that from the Langmuire model (*R*^2 = 0.946), which suggests that Freundlich isotherm model is more suitable to describe the adsorption process of Pd(II) ions on Pd(II) IIPs. Freundlich model is an empirical equation, it is generally believed that the reciprocal of the Freundlich constant *n* is negatively correlated with the adsorption performance [27]. When 1/*n* is between 0.1~0.5, it is easy to adsorb; when 1/*n* is greater than 2, it is difficult to adsorb. The 1/*n* value obtained from this experiment is about 0.39, which indicates that Pd(II) ions are easy to be adsorbed by Pd(II) IIPs.

In the kinetic study, the fitting result agrees better with the pseudo-second-order kinetic model (*R* ^2^ = 0.971) than with the pseudo-first-order kinetic model (*R* ^2^ = 0.896). With a theoretical equilibrium adsorption capacity of 5.085 mg/g which is closer to the experimental value of 5.042 mg/g, the adsorption of Pd(II) ions on Pd(II) IIPs is considered to be more in accordance with the pseudo-second-order kinetic model. The pseudo-second-order kinetic model assumes that the rate-controlling steps are mainly chemical adsorption processes between heavy metal ions and the adsorption sites on absorbents [31]. Therefore, the adsorption of Pd(II) ions on Pd(II) IIPs is mainly contributed by chemical reactions, thus confirming the formation of imprinted recognition sites.

## Conclusion

Studies on three kinds of functional monomers during the synthesis of Pd(II) IIPs showed significant different imprinting effects. The UV spectra showed that 4-VP and ANA caused a large structural change after the template reacted with three functional monomers, while AAA basically did not change. According to the batch adsorption experiments, 4-VP performed as the most promising candidate functional monomer with a higher Pd(II) adsorption capacity than ANA and AAA. Formation of imprinted recognition sites, which was beneficial to Pd(II) IIPs adsorption for Pd(II) ions, was evidenced by the FTIR spectra. At optimal working conditions, a theoretical equilibrium adsorption capacity of 5.085 mg/g was obtained for Pd(II) ions by the synthesized Pd(II) IIPs. Compared with Cu(II), Zn(II), Ni(II), and Pt(II), Pd(II) IIPs showed high selectivity for Pd(II) ions. The isothermal results suggested that the Freundlich isotherm model demonstrated a better fitting for the adsorption process of Pd(II) on Pd(II) IIPs than the Langmuir isotherm model. Kinetic studies illuminated that the adsorption process could be best described by the pseudo-second-order kinetic model.

## Abbreviations

4-VP: 4-Vinylpridine
AAA: 2-Acetamidoacrylic acid
ANA: 2-(allylthio)nicotinic acid
BET: (Brunauer, Emmett, Teller’s test)
FAAS: Flame atomic absorption spectrophotometry
FTIR: Fourier transform infrared spectroscopy
NIPs: Non-ion-imprinted polymeric nanospheres
Pd(II) IIPs: Palladium(II) ion-imprinted polymeric nanospheres
SEM: Field-emission scanning electron microscope
TGA: Thermogravimetry analyses
UV: Ultraviolet visible
